# Disgusted, but amused: positive emotion attenuates disgust elicited by film clips

**DOI:** 10.3389/fpsyg.2025.1565884

**Published:** 2025-05-14

**Authors:** Benjamin J. Mitchell, Karin G. Coifman

**Affiliations:** Psychological Sciences, Kent State University, Kent, OH, United States

**Keywords:** disgust, positive emotion, psychopathology, humor, exposure therapy

## Abstract

**Background:**

Disorders like obsessive-compulsive disorder are associated with heightened disgust. Research suggests that dominant methods for treating such disorders (e.g., exposure therapies) are less effective at targeting disgust. Alternative strategies are needed to enhance treatment effectiveness.

**Methods:**

In two studies, we investigated positive emotion (elicited via humorous content) for attenuating disgust responses to film clips. In Study 1, *n* = 174 undergraduates were randomized to view either a humorous, sad, or neutral clip prior to a disgusting clip. In study 2, *n* = 294 undergraduate participants were randomized to either view two clips with discrete emotional content (purely disgusting and purely amusing) or two mixed emotional clips (disgust mixed with amusement, amusement mixed with disgust).

**Results:**

Results of Study 1 showed that the humorous clip buffered against ratings of disgust. In Study 2, humorous content reduced reports of disgust. For both studies, the effect of the manipulation was not moderated by clinical characteristics, like disgust proneness, contamination concerns, or depression.

**Conclusion:**

Findings suggest that positive emotions can alter the appraisal of disgusting content, attenuating feelings of disgust, with potential clinical implications for treatment.

## Disgusted, but amused: positive emotion attenuates disgust elicited by film clips

Disgust is a basic universal emotion that evolved to motivate the avoidance of germs (Curtis and Biran, 2001; Oaten et al., 2009) and is associated with psychiatric conditions such as obsessive-compulsive disorder (OCD), specific phobias, and post-traumatic stress disorder (PTSD; Bhikram et al., 2017; de Jong et al., 2002; Knowles et al., 2018; Olatunji et al., 2010). Because these disorders have traditionally been conceptualized as disorders of fear (Olatunji and McKay, 2009; Mitchell and Olatunji, 2024), exposure therapies are the dominant treatment approach. However, disgust has been shown to be resistant to extinction (Mason and Richardson, 2012; Mitchell et al., 2024a) and habituation (Olatunji et al., 2009), rendering exposure interventions less effective at reducing disgust (Pascal et al., 2020). Emerging research suggests boosting positive emotions may assist in attenuating disgust (Deckman and Skolnick, 2021; Randler et al., 2016). The present investigation presents two investigations to further examine the impact of positive mood induction (via humorous content) on disgust in response to film clips, and we explore the clinical implications of the effects.

There are at least two clinical challenges associated with treating disgust responses. As noted, disgust is more resistant to extinction when compared to fear (i.e., meta-analytic evidence: Mitchell et al., 2024a), and habituation to disgusting stimuli occurs at a slower rate (Olatunji et al., 2009). Second, patients appear less willing to engage with disgusting stimuli, rendering exposure therapy for disgust-related disorders even more challenging (e.g., Jalal et al., 2022). Indeed, visual avoidance is an automatic response to disgusting stimuli that is difficult to attenuate (Mason and Richardson, 2012) and research applying behavioral tasks has documented the difficulty of high disgust-prone individuals in approaching disgusting stimuli (Goetz et al., 2013a; Goetz et al., 2013b; Koch et al., 2002). Relatedly, some patients simply refuse to engage in exposures during therapy, with high rates of dropout and refusal for contamination-related exposures (Najmi and Amir, 2010; Hezel and Simpson, 2019; Rachman et al., 2011). Importantly, Fink-Lamotte et al. (2020) found that participants’ reported willingness to engage in disgusting exposures was a primary predictor of disgust reduction during a laboratory exposure session. Thus, investigating novel methods for increasing behavioral approach toward disgusting stimuli will be an important step toward improving treatments for targeting disgust responses.

One promising method is the use of positive mood inductions. Positive emotions may operate as a regulatory resource (Fredrickson, 2001; Waugh, 2020), helping to “undo” the effects of negative emotion (Fredrickson and Levenson, 1998). For example, boosting positive emotion (often via humorous film clips) can reduce fear and anxiety (Abel, 1998; Ford et al., 2012). Specifically, humor interventions have been shown to boost positive emotion and reduce exam-specific fears and worry to the point of improving test performance (Berk and Nanda, 2006; Perlini et al., 1999). More recently, two studies have applied positive mood induction via humorous film clips to reduce disgust-linked avoidance behaviors suggesting clear relevance in treatment. Randler et al. (2016) found that presenting a humorous film clip to biology students immediately prior to a laboratory activity of dissecting a fish resulted in reduced anticipated disgust related to the exercise. In addition, Deckman and Skolnick (2021) found that individuals who viewed humorous film clips reported a greater willingness to engage in disgust-inducing activities (e.g., eating a bug or using a dirty toilet). However, findings are not always consistent. For example, Borg and de Jong (2012) investigated the effects of films clips on disgust-related avoidance and found that a sexually arousing clip, but not a generally “positive arousing” clip, reduced disgust-related avoidance. Notably, the positive clip used in this study was a “sports/high adrenaline” clip shown to an all-female sample, which may have been less effective at attenuating disgust than the humorous clips used in other studies (Deckman and Skolnick, 2021; Randler et al., 2016). Thus, humorous content might be the preferred source of positive emotion for targeting disgust-related processes.

There are a few plausible channels by which positive emotions elicited via humor (e.g., amusement) may be particularly effective at reducing disgust. First, humor may function as an emotion regulatory process (Saroglou and Anciaux, 2004), facilitating re-appraisal of aversive experiences (Samson et al., 2014) and reducing the impact of stressful events (Fritz et al., 2017). For example, appreciation for “sick humor” (e.g., disgusting jokes) is associated with the use of humor and emotional expression as coping strategies (Saroglou and Anciaux, 2004), which may be adaptive for confronting disgusting stimuli. Additionally, because amusement tends to facilitate an approach-oriented motivational tendency (Christie and Friedman, 2004), eliciting amusement prior to or simultaneously with disgust may help individuals overcome the motivation to avoid. Lastly, positive emotions can reduce autonomic arousal and related sensations that are a core feature of disgust. For example, positive emotions have been shown to increase parasympathetic nervous system activity (e.g., increasing heart-rate variability) which can help down-regulate sympathetic arousal typically associated with responses to threat (Kop et al., 2011). In addition, disgust has been shown to be associated with visceral, gastric reactivity and nausea (Shenhav and Mendes, 2014), and research has found that positive emotions can reduce nausea in individuals coping with illness (e.g., Chaves et al., 2016) and individuals undergoing disorienting and nausea-inducing activities (e.g., virtual reality immersion; Kaufeld et al., 2022). Thus, it is possible that positive emotions can help to reduce physical sensations, like nausea and arousal, during disgust reactions. Together, these findings suggest that integrating amusing content into disgust-laden contexts may have considerable impact, which could be an advantage for targeting disgust for clinical treatment purposes.

## The current investigation

We conducted two experiments investigating the effects of positive emotion on disgust responses. For Study 1, we tested whether participants randomized to receive a positive mood induction (via a humorous film clip) prior to viewing a disgusting film clip were buffered against subsequent disgust responses. For Study 2, we tested whether individuals randomized to view humorous content embedded *within* disgusting film clips was associated with attenuated disgust responses compared to those viewing purely disgusting film clips. For both studies, we used film clips taken from a standardized, validated set (Gilman et al., 2017). In Study 2, we manipulated two of the disgusting and humorous clips used in Study 1 to present mixed contexts (disgusting and amusing). For the disgusting clip, we edited in an audio laugh track to increase the positive appraisal of the clip. For the humorous clip, we edited in an audio track of vomiting noises to increase the disgust appraisal of the clip. Finally, for both studies, we explored whether any benefits from humorous content are maintained when exploring interactions with clinically relevant characteristics of the samples, including depression (Study 1 only), disgust proneness (both studies), and contamination-based OCD symptoms (Study 2 only). Depression often co-occurs with disgust-related pathology (e.g., OCD: Goodwin, 2015; PTSD: Rytwinski et al., 2013) and is broadly associated with reduced positive emotional reactivity (Rottenberg et al., 2005) possibly due to disruptions in reward systems (Admon and Pizzagalli, 2015). Therefore, any intervention aimed at manipulating positive emotion systems should be shown to be effective even for those less reactive to positive stimuli, and for those with higher disgust-related propensities (disgust proneness and contamination concerns). If a positive mood induction can be effective at buffering disgust responses even for individuals with heightened clinical characteristics, it may be a promising method for enhancing the effectiveness of treatments targeting disgust-related symptoms.

Although positive mood induction has been shown to reduce *anticipated* disgust (Randler et al., 2016) and facilitate greater *reported* willingness to approach disgusting things (Deckman and Skolnick, 2021), currently no research has investigated whether positive mood induction can directly buffer against and attenuate disgust responses when presented before or simultaneously with a disgusting stimulus. Thus, the current investigation aimed to conceptually replicate previous findings and extend the literature by exploring the clinical applicability of positive mood induction for attenuating disgust responses when considering clinical characteristics as moderators. All procedures were approved by the Kent State Institutional Review Board.

## Transparency and openness

Study 1 was not pre-registered, but the Study 2 data collection and analysis plan were pre-registered (https://osf.io/fwa84/?view_only=c257a87fbe24495f98c2fefdfa0c306c). The data and code used for analyses in both studies can be found at the following link: https://osf.io/89xtc/?view_only=057fac78a45f437abcc2d8330bad80fb. Sample size was determined for both studies using *a priori* power analyses conducted in G*Power 3.1.9.7 (Faul et al., 2009). For Study 1, the analysis indicated that a sample of *n* = 159 is required to detect a medium effect size with 80% power using One-way ANOVA with three groups. A medium effect size is consistent with prior related research (e.g., Randler et al., 2016). For Study 2, a sample of *n* = 98 is required to detect a medium effect size with 80% power using a between-subjects repeated measures ANOVA with two measurements. Again, a medium effect size is consistent with prior related research (e.g., Randler et al., 2016), as well as the results from Study 1 of the present investigation. For both studies, we had sufficient power for the primary analyses (Study 1: *n* = 174; Study 2: *n* = 294). The sample size for Study 2 is much larger than required because it was part of another, unrelated pre-registered study derived from the same data collection.

## Study 1: does positive mood induction buffer against disgust responses?

## Methods

### Participants

Two-hundred and fourteen undergraduate participants were recruited for an online study via the university’s subjects pool and were compensated with course credit. Thirty-eight participants failed at least one attention check question embedded in the study procedures (e.g., “Please select Strongly Agree”), and three of the remaining participants did not complete the experimental procedure, thus the final sample consisted of *n* = 174 participants (77.6% female). The final sample and those who were dropped from analyses did not significantly differ in age, *t*(209) = 1.69, *p* = 0.094, *d* = 2.79, but the final sample (*M* = 31.05, *SD* = 7.87) did have significantly higher disgust propensity than those who were dropped (*M* = 27.73, *SD* = 7.49), *t*(209) = 2.35, *p* = 0.020, *d* = 0.43. The sample mean age was 20.39 (*SD* = 3.02), with the majority (*n* = 143; 82.2%) identifying as White. See Supplementary Table S1 for a breakdown of the sample characteristics.

### Procedure

Participants were invited to participate in an online study, hosted via Qualtrics, investigating emotional responses to film clips. Participants provided informed consent and then completed several questionnaire measures, including demographics, personality, and measures of psychological symptoms. Of these measures, demographics (e.g., sex), a measure of disgust propensity, and depression symptoms were included in the current investigation. Then, participants were randomly assigned (via Qualtrics) to view one of three film clips varying in emotional valence (positive, negative, or neutral) prior to viewing a disgusting film clip. Specifically, participants either first viewed a validated positive film clip (eliciting positive emotion, e.g., amusement), a validated negative film clip (eliciting negative emotion, e.g., sadness), or a validated neutral film clip (Gilman et al., 2017). Following the first film clip, participants provided positive (e.g., enjoyment, amusement, etc.) and negative (e.g., fear, sadness, etc.) affect ratings. Next, all participants viewed the same film clip demonstrated to reliably elicit disgust (Gilman et al., 2017) and then completed the same emotion ratings. After providing affect ratings, each film clip was followed by a single-item multiple choice quiz question (e.g., “What is the primary setting of the film clip?”) to check for engagement. After completing the experimental procedures, participants viewed a short, mood lifting film clip, were invited to contact the lab with any questions, and were provided a downloadable list of campus and local mental health referrals. Importantly, data were collected in March – May 2020, during the early months of the COVID-19 pandemic (Cucinotta and Vanelli, 2020). At this time, all participants in the study had switched to online learning and stay-at-home orders were in place in the state where data collection occurred.

### Measures

*Disgust Scale Revised* (*DS-R;* Olatunji et al., 2007a): The DS-R was administered as a measure of individual differences in disgust propensity–the trait-like tendency to experience disgust across contexts. The DS-R contains two sections: First, participants rated their level of agreement with 13 items (e.g., “If I see someone vomit, it makes me sick to my stomach”) from 0 (strongly disagree) to 4 (strongly agree). Next, they rated how disgusting they viewed 12 scenarios (e.g., “You see maggots on a piece of meat in an outdoor garbage pail”) on a scale from 0 (not disgusting at all) to 4 (extremely disgusting). The DS-R contains three sub-scales, including core disgust (disgust in response to pathogen threats, such as bodily excretions), contamination disgust (disgust in response to potential contaminants, such as a contaminated beverage), and animal reminder disgust (disgust in response to reminders of our animal nature). For the current study, we considered the core disgust sub-scale as a covariate in our primary analyses (only if the experimental conditions differed) for its relevance to the content in the disgusting clip. We also explored it as a moderator of the effect positive mood induction on disgust reactions. Internal reliability was adequate (*𝛼* = 0.77), and mean core disgust was 31.05 (SD = 7.87), which is comparable to other undergraduate samples (Olatunji et al., 2012).

*Center for Epidemiological Studies – Depression* (*CES-D;* Radloff, 1977): The CES-D is a 20-item self-report index of depression symptoms. Participants rated statements about how they have been feeling during the past month (e.g., “I felt depressed”) on scale from 0 (rarely or none of the time) to 3 (most or all the time). Scores of 16 and above serve as a clinical cut-off, indicating high risk for clinical levels of depression (Lewinsohn et al., 1997). Internal consistency was excellent (*α* = 0.93). The current sample reported relatively high levels of depression symptoms (*M* = 19.55, *SD* = 11.98), which may be due to the disruption of the COVID-19 pandemic, as depression rates rose substantially in college students (Fruehwirth et al., 2021).

*Film Clips:* During the experimental procedure, participants were randomized to view one of three film clips (positive, negative, or neutral) prior to viewing a disgusting film clip. The positive clip was a compilation of *Funny Cats* home video, which has been shown to elicit amusement and happiness (Gilman et al., 2017). The negative film clip was the final scene from the movie, *The Champ*, in which a young boy cries as his father passes away from injuries sustained in a boxing match, which reliably elicits sadness (Gilman et al., 2017; Rottenberg et al., 2007). We chose a sad clip because sadness has a negative valence consistent with disgust, but has lower levels of arousal (Russell, 2003), and does not covary with disgust to the same degree as other self-reported negative emotions (e.g., fear; Olatunji and Sawchuk, 2005). We used a clip from *Alaska’s Wild Denali*, a documentary film, as the neutral film clip, which does not elicit strong positive or negative emotions (Gilman et al., 2017). After participants viewed their randomly assigned film clip, all participants viewed a clip from the movie *Trainspotting*, which has been shown consistently to elicit high levels of disgust with relative specificity and discreteness (Gilman et al., 2017). The clip depicts a man defecating in a grotesque bathroom (covered in feces) and eventually falling into the toilet. All film clips were approximately five minutes long. Of the 174 participants, *n* = 60 were randomized received the positive film, *n* = 59 received the negative film, and *n* = 55 were in the neutral prime condition.

*Affect:* Following the first (randomly assigned) clip and then after the disgusting clip, participants rated how they felt using a list of emotion words on a scale from 1 (none) to 7 (strong). The list included interest, fear, relief, sadness, enjoyment, distress, surprise, guilt, happiness, anger, amusement, disgust, affection, boredom, and fatigue, consistent with prior research (including the film clip validation studies: Gilman et al., 2017) reflecting varying levels of arousal and valence per dominant models of affect (Rafaeli et al., 2007). For the primary outcome measure, we focused on ratings of disgust in response to the disgusting clip. In addition, we computed positive affect (mean of all positive emotion words; interest, relief, enjoyment, happiness, amusement, and affection) and negative affect (mean of all negative emotion words; fear, sadness, distress, guilt, anger, and disgust) scores for all film clips. Surprise, boredom, and fatigue were excluded, as in prior research, due to their ambiguous valence.

### Data analytic plan

As preliminary analyses, we used one-way analysis of variance (ANOVA) and Chi-Square to verify that the three conditions had equivalent sample characteristics (e.g., demographics, depression, and core disgust propensity). In addition, we conducted a manipulation check via two one-way ANOVAs to ensure the positive film clip elicited significantly higher positive affect than the negative and neutral clips, and that the negative film clip elicited significantly higher negative affect than the positive and neutral clips.

For primary analyses, we conducted one-way ANOVA to test for group differences (by condition) in disgust ratings in response to the disgusting film clip. We followed the main effect of group with post-hoc pairwise comparisons to determine whether the positive condition had significantly lower disgust ratings compared to the negative and neutral conditions. Next, we re-ran the main ANOVA analysis substituting disgust with negative affect (in response to the disgust film clip) to determine whether the effect of the positive mood induction reduced negative emotions more generally. As exploratory analyses, we ran regression analyses with moderation to determine if the effect of the positive clip depended on depression symptoms or disgust propensity.

## Results

### Preliminary analyses

First, one-way ANOVA and Chi-Squared analyses showed no difference by randomized group in core disgust propensity, age, sex, race, and ethnicity based on condition (see Supplementary Table S1). Next, a one-way ANOVA testing for group differences in positive affect following the initial film clip confirmed a main effect of group (after applying Brown-Forsythe corrections for unequal variances), *F*(2,141.76) = 22.15, *p* < 0.001, *η_p_^2^* = 0.21, with Games-Howell post-hoc comparisons demonstrating that the positive clip (*M* = 3.55, *SD* = 1.56) elicited significantly higher levels of positive affect than the negative (*M* = 2.02, *SD* = 0.80, *p* < 0.001, *d* = 1.24) and neutral (*M* = 2.72, *SD* = 1.41, *p* = 0.006, *d* = 0.58) clips. In addition, the neutral clip elicited significantly higher positive affect than the negative clip (*p* = 0.003, *d* = 0.44). Results from the second one-way ANOVA also confirmed a main effect of group (with Brown-Forsythe corrections) for negative affect, *F*(2,109.76) = 90.62, *p* < 0.001, *η_p_*^2^ = 0.51, with Games-Howell comparisons demonstrating that the negative clip (*M* = 3.15, *SD* = 1.28) elicited significantly higher negative affect than the positive (*M* = 1.27, *SD* = 0.56, *p* < 0.001, *d* = 1.90) and neutral (*M* = 1.19, *SD* = 0.64, *p* < 0.001, *d* = 1.94) clips. The positive and neutral clips did not significantly differ in negative affect (*p* = 0.754, *d* = 0.13). See Supplementary Figure S1a and Supplementary Figure S1b in the Supplementary material for plotted results.

### Primary analyses

**Figure 1:**
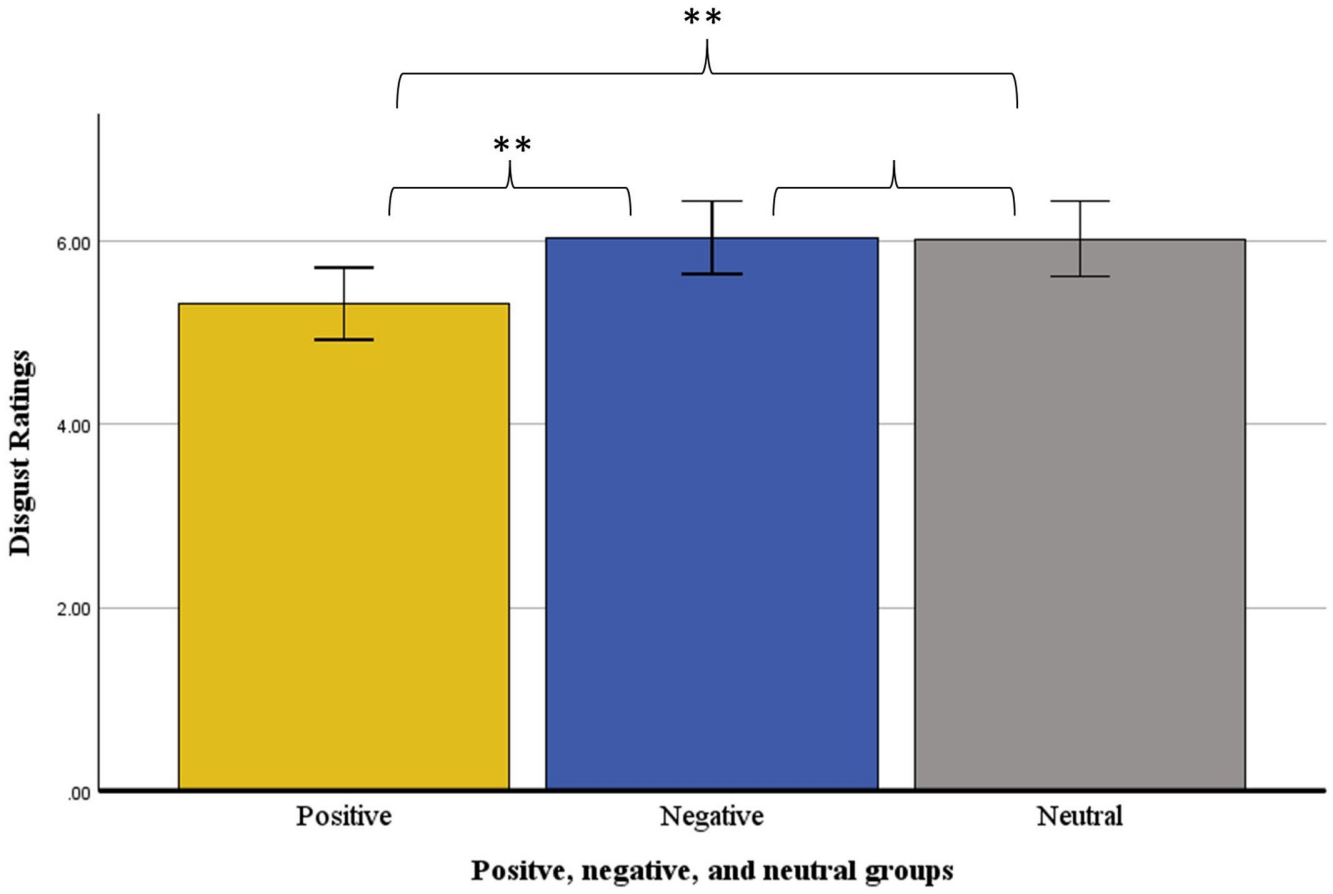
Group differences in disgust ratings following the disgust film clip (Study 1).

ANOVA was used to test for group differences in disgust reactions to the disgusting film clip. Results showed a main effect of group, *F*(2,171) = 4.15, *p* = 0.017, *η_p_*^2^ = 0.05. Pairwise comparisons using estimated marginal means showed that participants in the positive group (*M* = 5.32, *SE* = 0.20) reported significantly lower levels of disgust than the negative (*M* = 6.03, *SE* = 0.20, *p* = 0.012, *d* = 0.44) and neutral (*M* = 6.02, *SE* = 0.21, *p* = 0.016, *d* = 0.46) groups. The negative and neutral groups did not significantly differ in ratings of disgust (*p* = 0.957, *d* = 0.01). See Figure 1 for the plotted results.

To test whether the effects of the positive mood induction generalized to overall negative affect, we re-ran the ANOVA with overall negative affect in response to the disgusting film clip as the dependent variable. Results showed a main effect for group, *F*(2,171) = 4.13, *p* = 0.018*, η_p_^2^* = 0.05. Pairwise comparisons using estimated marginal means showed that participants in the positive group (*M* = 2.38, *SE* = 0.12) reported significantly lower levels of negative affect than both the negative (*M* = 2.79, *SE* = 0.12, *p* = 0.013, *d* = 0.47) and neutral (*M* = 2.79, *SE* = 0.12, *p* = 0.016, *d* = 0.48) groups. The negative and neutral groups did not significantly differ in negative affect (*p* = 0.971, *d* = 0.01).

### Exploratory analyses

Next, we explored whether the positive mood induction remained effective for those with clinically elevated disgust propensity and depression. To do this, we dummy coded group so that the neutral and negative groups were coded as 0 and the positive group was coded as 1. Then we ran two regression models and tested disgust propensity and depression as continuous moderators of the relationship between group and disgust ratings. For the first model, there was no group by disgust propensity interaction (*B* = −0.02, *p* = 0.425). For the second model, there was, again, no group by depression symptoms interaction (*B* = −0.00, *p* = 0.967).

## Study 1 discussion

Results from Study 1 suggest that a positive mood induction, via a humorous film clip, buffers against subsequent disgust responses when compared to a sad or neutral film clip. Moreover, the effect did not depend on level of disgust propensity or depression symptoms, suggesting it may have utility in a clinical context. In addition to buffering against disgust, specifically, the positive mood induction buffered against overall reported negative affect. Thus, positive mood inductions may broadly reduce subjective reports of disgust and negative affect in response to disgusting stimuli.

## Study 2: does positive content *within* a disgusting stimulus attenuate self-reported disgust?

In Study 2, we aimed to determine if presenting a disgusting video with embedded humorous content would lead to lower disgust ratings compared to a solely disgusting film clip. Thus, in contrast to Study 1 where the positive mood induction preceded the disgusting clip, in Study 2, the humorous and disgusting content were presented simultaneously. From a clinical perspective, a positive mood induction prior to exposure might encourage initial engagement, while injecting humor during an exposure might help maintain engagement and facilitate cognitive reappraisal. We presented mixed content (disgusting and amusing) in two ways: (1) amusing content (laugh track) edited into a primarily disgusting clip and (2) disgusting content (vomit sounds) edited into a primarily humorous clip. Although we are primarily interested in the impact of the positive content on the disgusting clip, examining the reverse allows us to document the difference in impact of humorous versus disgusting content. The data collection and analyses were pre-registered (https://osf.io/fwa84/?view_only=c257a87fbe24495f98c2fefdfa0c306c) and occurred in Fall, 2023.

## Methods

### Participants

Undergraduate participants (*n* = 377) were recruited for an online study via the university’s subject pool and were awarded course credit. Participants who failed at least one attention check question embedded within the study procedures (e.g., “Please select Strongly Agree”) and were removed (*n* = 83), leaving a final sample of *n* = 294 participants. Mean age was 19.98 (*SD* = 3.53), and the sample was majority female (*n* = 227; 77.2%) and majority white (*n* = 243; 82.7%). See Supplementary Table S2 for sample characteristics. The final sample did not significantly differ from those who were dropped for age, *t*(359) = 0.95, *p* = 0.341, or disgust propensity, *t*(361) = −0.53, *p* = 0.594, but, the final sample (*M* = 9.69, *SD* = 7.028) had lower reported contamination concerns than those who were dropped (*M* = 12.08, *SD* = 6.68), *t*(363) = −2.60, *p* = 0.005, *d* = 0.34.

### Procedure

The link to the study (hosted via Qualtrics) was listed via the university’s research subjects pool website, where participants were invited to participate in an online study (called “Mixed Emotions in Film”) investigating mixed emotional responses to film clips in exchange for course credit. After providing informed consent, participants completed several questionnaire measures, including demographics, personality, and measures of various psychological symptoms. Only demographics, disgust propensity, and contamination-based OCD symptoms were included in the present investigation. Next, participants engaged in the experimental video task. First, all participants viewed a neutral, baseline clip. Then, participants were randomized into one of two conditions: *discrete* film clips or *mixed* film clips. Those in the discrete clips group viewed an unaltered disgusting clip (same clip as Study 1; *Trainspotting*) and a humorous clip (same as Study 1; *Funny Cats*). The mixed clips group viewed the same disgusting clip, but with a laugh track edited into the audio to elicit mixed emotions (disgust and positive affect). Those in the mixed group also viewed the same humorous clip, but with vomit sounds edited into the audio (again, to elicit both positive affect and disgust). Following each clip, participants provided affect ratings, and then responded to a single-item multiple choice quiz question (e.g., “What is the primary setting of the film clip?”) to check for engagement. Finally, participants viewed a short, mood lifting film clip, were invited to contact the lab with any questions, and were provided a downloadable list of campus and local mental health referrals.

### Measures

*Disgust Scale Revised* (*DS-R;* Olatunji et al., 2007a): The core disgust propensity subscale was included in exploratory analyses as a moderator of the relationship between the experimental condition and disgust ratings in response to the disgusting film clip. Internal consistency was adequate (*α* = 0.74). Mean core disgust propensity was 33.61 (*SD* = 7.60), which is comparable to Study 1.

*Obsessive-Compulsive Inventory-Revised Washing Subscale (OCI-R Washing*; Foa et al., 2002): The OCI-R Washing subscale was used to index contamination-based OCD symptoms and was explored as a moderator of the effect of positive content on disgust responses. The washing subscale includes 9 items (e.g., “I wash and clean obsessively”) rated on a Likert scale from 0 (Not at all) to 4 (Extremely). Internal consistency was good (*α* = 0.89), and mean washing symptoms (*M* = 9.69, SD = 7.03) were similar to other undergraduate samples from past research (e.g., Thorpe et al., 2011).

*Film Clips:* The initial neutral clip was a clip from *Alaska’s Wild Denali*, which was used as the neutral clip in Study 1. The *Trainspotting* film clip (used in Study 1) was included as the disgusting clip for the both the discrete and mixed conditions. However, for the mixed condition, we added an audio laugh track to elicit both disgust and positive affect. Because laughter is thought to be a social phenomenon (Scott et al., 2014), research has shown that laugh tracks encourage laughter and can induce positive affect (Gillespie et al., 2016; Kanthan et al., 2016). Lastly, the *Funny Cats* clip (from Study 1) was used as the humorous clip for both conditions, but for the mixed condition, audio clips of vomiting noises were edited into the video to induce disgust. All film clips were approximately five minutes in length, and the two primary clips were randomized within-person.

*Affect:* We used the same affect words list as Study 1, but we added two additional disgust-related words (nausea and revulsion) to derive a disgust response based on a three-item composite (mean of the three ratings). These additional words were derived from the Discrete Emotions Questionnaire for a multi-item index of state disgust (Harmon-Jones et al., 2016). Internal consistency of the disgust composite was good for each condition and across all clips (Discrete Disgust Clip: *α* = 0.76; Discrete Humorous Clip: *α* = 0.72; Mixed Disgust Clip: *α* = 0.80; Mixed Humorous Clip: *α* = 0.91). We measured negative affect using the same words as Study 1. Internal consistency was good across conditions and clips (Discrete Disgust Clip: *α* = 0.79; Discrete Humorous Clip: *α* = 0.81; Mixed Disgust Clip: *α* = 0.81; Mixed Humorous Clip: *α* = 0.88). We measured positive affect using the same words as Study 1. Internal consistency for positive affect ratings were also good across conditions and clips (Discrete Disgust Clip: *α* = 0.75; Discrete Humorous Clip: *α* = 0.91; Mixed Disgust Clip: *α* = 0.79; Mixed Humorous Clip: *α* = 0.89).

### Data analytic strategy

In our preregistration, we hypothesized, based on Study 1, that when a disgusting clip had amusing content added (via a laugh track), participants would report higher positive affect and lower disgust than those watching the discretely disgusting clip. In addition, we expected that when a humorous clip had disgusting content added (via vomit sounds), participants would report lower positive affect and higher disgust than those watching the discretely humorous clip. To test these hypotheses, we first conducted manipulation checks by running paired-samples t-tests comparing disgust and positive affect ratings for each clip with ratings for the baseline neutral clip. Next, as primary analyses, we conducted three 2 × 2 (Group: Discrete vs. Mixed; Clip: disgust and humorous) repeated measures ANOVAs to examine group differences in disgust ratings, negative affect ratings, and then positive affect ratings for the clips. For each ANOVA, we conducted post-hoc comparisons to explicitly test if: (a) The Mixed (versus discrete) group reported lower disgust levels and higher positive affect in response to the disgust-inducing clip, and (b) The Mixed group (versus discrete) reported higher disgust levels and lower positive affect in response to the humorous clip. Lastly, we explored whether the attenuating impact of positive content in the disgusting clip were present in individuals with elevated core disgust propensity, contamination-based OCD symptoms, and depression. To do this, we ran two linear regression models with group as the predictor, disgust ratings for the disgusting clip as the outcome variable, and either disgust propensity or contamination-based OCD symptoms as the moderators (ran in separate models).

## Results

### Preliminary analyses

First, independent-samples t-tests and Chi-Square analyses showed no group differences in core disgust propensity, contamination-based OCD, age, sex, race, and ethnicity based on condition (see Supplementary Table S2 for a full breakdown of demographics with comparisons between conditions). Manipulation checks via paired-samples t-tests confirmed that within the discrete condition sample, the discrete disgust clip (*M* = 4.90, *SD* = 1.77) elicited higher disgust ratings than the baseline clip (*M* = 1.25, *SD* = 0.79), *t*(143) = −22.28, *p* < 0.001, *d* = 1.86, but the baseline neutral clip and the discrete humorous clip (*M* = 1.14, *SD* = 0.47) did not significantly differ for disgust ratings, *t*(143) = 1.93, *p* = 0.056. In addition, positive affect ratings for the discrete humorous clip (*M* = 4.01, *SD* = 1.56) were higher than the baseline clip (*M* = 3.12, *SD* = 1.33), *t*(143) = −6.96, *p* < 0.001, *d* = 0.58, and they were lower for the discrete disgust clip (*M* = 1.73, *SD* = 0.86), *t*(143) = 10.89, *p* < 0.001, *d* = 0.91. Within the mixed condition sample, disgust ratings were higher for both the mixed disgust clip (*M* = 4.26, *SD* = 1.97), *t*(149) = −18.16, *p* < 0.001, *d* = 1.48, and mixed humorous clip (*M* = 2.90, *SD* = 2.10), *t*(149) = −18.16, *p* < 0.001, *d* = 0.74, compared to the neutral clip (*M* = 1.26, *SD* = 0.65). In addition, positive affect was lower for both the mixed disgust (*M* = 1.77, *SD* = 0.88), *t*(149) = 11.59, *p* < 0.001, *d* = 0.95, and mixed humorous clip (*M* = 2.93, *SD* = 1.59), *t*(149) = 2.31*, p* = 0.022, *d* = 0.19, compared to the neutral clip (*M* = 3.28, *SD* = 1.48). These results confirmed the clips had the intended effects on all participant ratings regardless of condition.

### Primary analyses

The first 2 × 2 repeated measures ANOVA included disgust ratings as the dependent variable. Results showed a significant between-subjects effect of group (discrete versus mixed), *F*(1,292) = 12.10, *p* < 0.001, *η_p_^2^* = 0.04. Post-hoc comparisons indicated that participants in the mixed condition (*M* = 4.26, *SE* = 0.15) reported lower disgust in response to the disgusting clip compared to those in the discrete condition (*M* = 4.90, *SE* = 0.16), *t*(292) = 2.92, *p* = 0.004, *η_p_^2^* = 0.03. Additionally, those in the mixed condition (*M* = 2.90, *SE* = 0.13) reported higher disgust in response to the humorous clip compared to those in the discrete condition (*M* = 1.14, *SE* = 0.13), *t*(292) = −9.82, *p* < 0.001, *η_p_^2^* = 0.25. Thus, the presence of the laugh track attenuated disgust appraisals of the disgusting clip, and the vomit track heightened disgust appraisals of the humorous clip.

Like Study 1, we re-ran this model with negative affect as the dependent variable. There was a significant main effect of group, *F*(1,292) = 8.16, *p* = 0.005, *η_p_^2^* = 0.03. Post-hoc comparisons were consistent with those for disgust. Participants in the mixed condition (*M* = 2.86, *SE* = 0.08) reported lower overall negative affect in response to the disgusting clip compared to those in the discrete condition (*M* = 3.24, *SE* = 0.10), *t*(292) = 2.71, *p* = 0.007, *η_p_^2^* = 0.02. In addition, those in the mixed condition (*M* = 2.16, *SE* = 0.08) reported higher negative affect in response to the humorous clip than those in the discrete condition (*M* = 1.19, *SE* = 0.08)*, t*(292) = −8.51, *p* < 0.001, *η_p_^2^* = 0.20.

**Figure 2:**
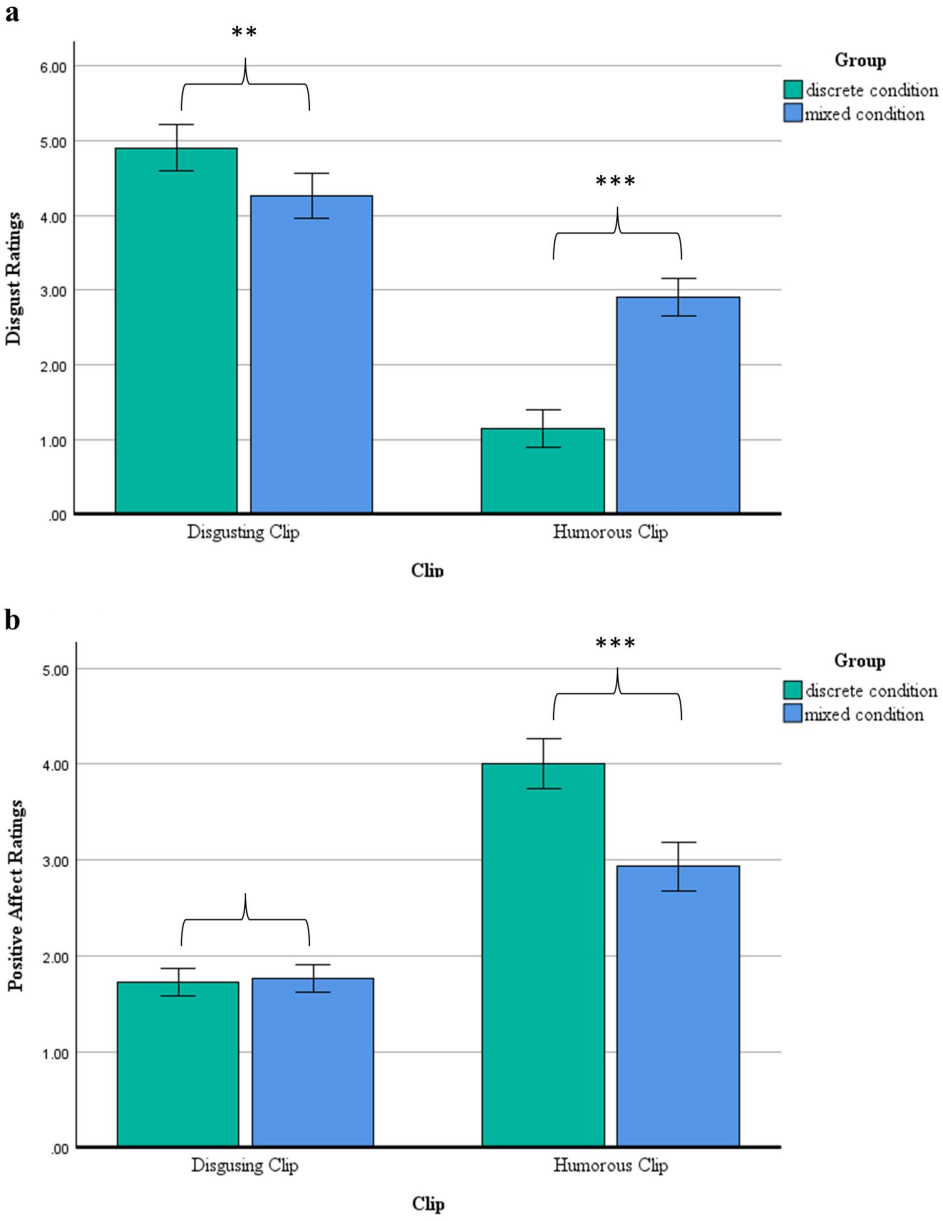
**(A)** Disgust ratings following the disgusting and humorous clips by condition (discrete vs. mixed) (Study 2). **(B)** Positive affect ratings following the disgusting and humorous clips by condition (discrete vs. mixed) (Study 2).

We re-ran this model with positive affect as the dependent variable. Again, results showed a significant main effect of group, *F*(1,292) = 21.08, *p* < 0.001, *η_p_^2^* = 0.07. Surprisingly, post-hoc comparisons showed no significant difference in reports of positive affect between the mixed and discrete groups in response to the disgusting clip, *t*(292) = −0.42, *p* = 0.69. However, those in the mixed group reported lower positive affect (*M* = 2.93, *SE* = 0.13) in response to the humorous clip compared to the discrete group (*M* = 4.01, *SE* = 0.13), *t*(292) = 5.84, *p* < 0.001, *η_p_^2^* = 0.11. Thus, the laugh track did not appear to explicitly increase positive affect in response to the disgusting clip, but the vomit track did reduce positive affect in response to the humorous clip (Figure 2).

### Exploratory analyses

We ran linear regression models with moderation to determine whether the attenuating effect of the positive content on disgust in response to the disgusting video depended on disgust propensity or contamination-based OCD symptoms. Results indicated there was no significant group by disgust propensity interaction (*B* = −0.02, *p* = 0.569), nor was there a significant group by contamination-based OCD symptoms interaction (*B* = 0.02, *p* = 0.589).

## Study 2 discussion

Results from Study 2 suggest that disgusting stimuli manipulated to include positive content *within* them are viewed as less disgusting than discretely disgusting stimuli. Moreover, this effect was not moderated by higher disgust propensity nor contamination-based OCD symptoms. This suggests that positive content artificially embedded within disgust content appears to be effective for individuals with elevated disgust propensity and contamination-based OCD.

## General discussion

Past research has shown that humorous film clips can attenuate anticipatory disgust (i.e., Randler et al., 2016) and reported willingness to engage in gross activities (Deckman and Skolnick, 2021). Across two studies, we further investigated whether positive emotion elicited via humorous content directly attenuates disgust in response to validated stimuli. In Study 1, by first boosting positive mood via a humorous film clip, participants rated a *subsequent* disgusting film clip as less disgusting than participants who first viewed a negative or neutral film clip, suggesting a clear buffering effect. In Study 2, a disgusting film clip with a laugh track embedded was rated as less disgusting than the same clip without the laugh track viewed by other participants. When we explored if these effects were moderated by key clinical indicators such as depression symptoms, trait disgust propensity or OCD symptoms, we found no evidence of moderation. These findings further support the use of positive emotion to reduce disgust toward film stimuli and suggest a potential novel pathway for clinical interventions targeting disorders where disgust is prominent.

Disgust has been studied through an evaluative conditioning framework, where neutral stimuli acquire disgusting qualities through their association with inherently disgusting stimuli (Olatunji et al., 2007b). Pairing a neutral conditioned stimulus (CS) with a disgusting unconditioned stimulus (US) changes the valence of the CS from neutral to disgusting. These learned disgust associations are notably resistant to extinction (Mitchell et al., 2024a). Based on the evaluative conditioning model of disgust, researchers have suggested that techniques aiming at altering the emotional valence of disgusting stimuli through positive emotional content might be most effective for interventions aimed at reducing disgust. For example, Ludvik et al. (2015) suggested that stimulus re-valuation techniques, like pairing a naturally disgusting stimulus (or unconditioned stimulus) with a positive stimulus, might be a promising technique for attenuating disgust acquired to neutral stimuli (e.g., conditioned disgust responses). Although not tested within a learning framework, results from this investigation are consistent with these ideas and demonstrate that highly disgusting content is viewed as less aversive when paired with humorous content.

In Study 2, we were able to clearly demonstrate that humorous content (in this case a laugh track) altered the appraisal of the disgust clip. Interestingly, this attenuating effect was present without an explicit increase in positive affect. It is possible that this change in disgust appraisals might have occurred through more implicit regulatory channels. Prior research has demonstrated that positive emotions can facilitate reduced autonomic arousal (e.g., Kop et al., 2011) and other aversive responses seen in disgust (e.g., nausea: Chaves et al., 2016; Kaufeld et al., 2022) which might explain the present findings. However, further investigation of this effect is needed to draw conclusions. To better account for the impact of these manipulations on positive mood and disgust, future replications of this investigation should also record participants’ broader bodily reactions, coding for smiles and laughter and indexing autonomic nervous system activity to better determine how the positive content might be influencing broader disgust reactions to these stimuli.

Importantly, the effects of adding the laugh track in Study 2 paled in comparison to the effect of adding the vomit track. The effect sizes were substantially different, and there was clear evidence that adding the vomit track increased reports of disgust and reduced positive affect, whereas adding the laugh track reduced reports of disgust but did not impact reports of positive affect. There are several ways to understand this finding. Disgust is a highly visceral emotion associated with high levels of autonomic arousal (Shenhav and Mendes, 2014), and therefore its presence in the mixed humorous stimulus likely overshadowed the positive content to some degree. In addition, disgust responses are thought to be acquired through evaluative conditioning, wherein properties from a disgusting stimulus can transfer over and contaminate neutral stimuli (Reynolds and Askew, 2021). Therefore, by adding the vomit audio track to the humorous clip, the clip became effectively *contaminated*, resulting in diminished positive affect and increased disgust. Although positive content can reduce disgust through emotion regulatory mechanisms, it seems that attenuating disgust is more challenging than the reverse: impacting positive emotions via disgust-inducing stimuli. This could be due to different levels of intensity and arousal associated with disgust versus positive emotions but could also be due to the primacy of negative interpretive/attentional biases, in which negative stimuli tend to capture greater attention than positive stimuli (Vaish et al., 2008). Although disgust had a larger negative effect on positive emotion, the effect of positive emotion on disgust is still clinically meaningful with potential utility in a treatment context.

Results have meaningful clinical implications for treating disgust-related disorders. Our explicit tests of moderation by common symptom dimensions suggest that the beneficial impact of positive emotions on disgust may persist even in clinical samples where positive emotional reactivity can be reduced – and – negative affectivity increased. For example, in a clinical setting, boosting an apprehensive patient’s mood prior to a session of disgust-related exposure therapy may boost initial engagement, while adding humor during an exposure might maintain engagement and contribute to beneficial cognitive changes (e.g., reappraisal). Indeed, a well-trained therapist might be able to use humor as a form of encouragement prior to and during initial exposure sessions, which may enhance behavioral approach and/or supply additional regulatory resources to overcome the challenge of disgusting exposures. Therapists might also find ways to make the exposure stimuli more humorous. Therapists’ use of humor has been shown to be associated with increased therapy effectiveness in general (Panichelli et al., 2018) and could be used strategically in this context. However, much more research is needed to explore the use of humor during disgust-related exposures, and standardized approaches should be considered. While most clinicians endorse the use of humor during psychotherapy, it is essential that it be used skillfully and cautiously (Hussong and Micucci, 2021). Humor may be inappropriate and insensitive in some contexts, such prolonged exposure treatment in a survivor of sexual violence where self-directed disgust is present (Jones et al., 2020).

A growing line of clinical research has shifted the focus from explicitly targeting negative reactions (e.g., exposure) and instead has started to emphasize the enhancement of positive emotions. For example, randomized clinical trials have found that “Positive Affect Treatment” aimed at increasing the frequency of positive emotions in individuals with depression and anxiety disorders was more effective at increasing positive affect and decreasing negative affect than a treatment focused on reducing negative emotions (Craske et al., 2023; Craske et al., 2019). Indeed, the frequency at which individuals experience positive emotions has been shown to be broadly and consistently associated with psychological health and wellbeing (Diener et al., 2009; Fredrickson and Joiner, 2018), and more persistent reports of positive emotions in daily life protect against psychological symptoms and loneliness 6–12 months later in high-risk groups (Mitchell et al., 2024b; Coifman et al., 2021). Given the challenge of treating disgust-related disorders with more traditional approaches (e.g., exposure therapies; McKay, 2006; Mitchell et al., 2024a), results from the present study suggest positive emotions can have a positive influence on disgust appraisals and could be an important target to enhance treatment effectiveness. Moreover, research should continue investigating the impact of positive emotions on disgust and disgust-related behaviors using both experimental research in laboratory, and more intense sampling approaches measuring these processes in daily life (e.g., ecological momentary assessment) in clinical samples. Research along these lines will help further ascertain the extent to which positive emotions might be clinically relevant for treating disgust-related symptoms and disorders.

The current investigation has considerable strengths. First, both studies consisted of randomized between-subjects experimental designs, allowing for causal inferences. Moreover, both studies used validated film clips from a standard set commonly used in affective science (e.g., Gilman et al., 2017). In addition, we used the results from Study 1 to pre-register the hypotheses, data collection, and analyses for Study 2. Finally, moderation analyses indicated the effects of the positive emotion manipulations from both studies did not appear to depend on the level of disgust proneness, depression symptoms, or OCD symptoms reported by the participants. Thus, these simple manipulations of positive mood appeared effective even for those with higher levels of underlying trait vulnerability to disgust-related disorders (disgust propensity, OCD), and those typically less reactive to positive emotional content (depression; Benning and Ait Oumeziane, 2017; Rottenberg et al., 2005). These results provide additional promise for targeting clinically significant disgust responses with positive emotion.

The results of both studies should be considered in light of their limitations. Both studies relied exclusively on self-report ratings of emotion for their outcome measures. Relatedly, there were inherent demand characteristics associated with the film clip tasks that may have influenced the direction of participants’ affect ratings (Lench et al., 2011). However, despite directional expectations (e.g., rating disgust high for clips with an explicitly negative valence), participants were blind to the experimental procedures and were unlikely to detect the full aims of the research. Whereas more objective behavioral indicators of emotion are ideal, self-report ratings provide meaningful information about the conscious appraisals of participants’ emotional experiences. Considering the promising trajectory of this emerging area (including the present investigation and past findings: Deckman and Skolnick, 2021; Randler et al., 2016), future research should expand on these findings by incorporating behavioral paradigms with additional, more objective measures (e.g., facial expressions, behavioral approach indices, etc.) to further explicate the impact of positive mood inductions on disgust-related phenomena. Another limitation is that the current investigation consisted of convenience samples of undergraduate students with limited diversity. Both studies consisted of majority female, undergraduate samples lacking in racial and ethnic diversity and future research should seek more diverse samples.

## Conclusion

Until recently, disgust has been a much-overlooked emotion in the study of psychopathology (Olatunji and McKay, 2009). There are unique, clinical challenges to targeting disgust in treatment, such as high refusal/dropout rates for exposure therapies (Najmi and Amir, 2010; Hezel and Simpson, 2019; Rachman et al., 2011) and disgust’s increased resistance to extinction (Mitchell et al., 2024a) and habituation (Olatunji et al., 2009) relative to fear. Thus, alternative methods for enhancing treatments are needed. Across two investigations, humorous content was shown to attenuate disgust reactions. First, experimentally manipulating positive mood via a humorous film clip was shown to buffer against disgust reactions to a subsequent disgusting film clip. Second, the presence of positive content (laugh track) within a disgusting clip was shown to attenuate the appraisal of disgust compared to a version of the clip without the positive content. For both studies, the effects were not moderated by relevant clinical characteristics (elevated disgust propensity, contamination-based OCD, depression). Results suggest that positive mood induction via humorous content is a promising direction for research on targeting clinically significant disgust responses.

## Data Availability

The datasets presented in this study can be found in online repositories. This data can be found here: https://osf.io/89xtc/?view_only=057fac78a45f437abcc2d8330bad80fb.
