# Supplementary material for: Disgusted, but amused: positive emotion attenuates disgust elicited by film clips
Source: Front Psychol. 2025 May 14;16:1565884. doi: 10.3389/fpsyg.2025.1565884 (PMC12116544; doi:10.3389/fpsyg.2025.1565884)
Supplement: Supplementary file 1 [file Supplementary_file_1.docx]

**Supplemental Material For:**

Disgusted, but amused: Positive emotion attenuates disgust reactivity in response to disgusting film clips

Data and Syntax Available at the OSF Project page: <https://osf.io/89xtc/?view_only=057fac78a45f437abcc2d8330bad80fb>

1. **Table S1**: ***Sample Characteristics by Condition (Study 1).* (p. 2)**
2. **Table S2: *Sample Characteristics by Condition (Study 2).* (p. 3)**
3. **Figure S1a: *Positive Affect Manipulation Check (Study 1)* (p. 4)**
4. **Figure S1b: *Negative Affect Manipulation Check (Study 1)* (p. 4)**

| Table S1: *Sample Characteristics by Condition (Study 1).* | | | | | | | |
| --- | --- | --- | --- | --- | --- | --- | --- |
|  |  | Positive Condition (*n* = 60) | Negative Condition (*n* = 59) | Neutral Condition (*n* = 55) | *F* | *χ^2^* | p-value |
| Age |  |  |  |  | 0.26 |  | .768 |
|  | *M* | 20.28 | 20.27 | 20.64 |  |  |  |
|  | *SD* | 2.78 | 3.16 | 3.15 |  |  |  |
| Core Disgust Propensity | |  |  |  | 0.51 |  | .601 |
|  | *M* | 30.27 | 31.22 | 31.73 |  |  |  |
|  | *SD* | 8.65 | 6.91 | 8.02 |  |  |  |
| Depression Symptoms | |  |  |  | 0.35 |  | .704 |
|  | *M* | 20.27 | 19.83 | 18.45 |  |  |  |
|  | *SD* | 11.11 | 11.81 | 13.16 |  |  |  |
| Sex Assigned at Birth | |  |  |  |  | 0.86 | .652 |
|  | Female | 45 (75%) | 45 (76.3%) | 45 (81.8%) |  |  |  |
|  | Male | 15 (25%) | 45 (23.7%) | 10 (18.2%) |  |  |  |
| Race |  |  |  |  |  | 5.70 | .680 |
|  | White/Caucasian | 49 (81.7%) | 47 (76.3%) | 47 (85.5%) |  |  |  |
|  | Black/African American | 8 (13.3% | 6 (10.2%) | 5 (9.1%) |  |  |  |
|  | Asian | 1 (1.7%) | 1 (1.7%) | 1 (1.8%) |  |  |  |
|  | American Indian/Alaska Native | 1 (1.7%) | 0 (0%) | 0 (0%) |  |  |  |
|  | Other | 1 (1.7%) | 5 (8.5%) | 2 (3.6%) |  |  |  |
| Ethnicity |  |  |  |  |  | 0.27 | .875 |
|  | Hispanic/Latino | 2 (3.3%) | 3 (5.1%) | 2 (3.6%) |  |  |  |
| Note: *n* = sample size; *M* = mean; *SD* = standard deviation; *F* = *F*-test statistic for one-way analysis of variance; *χ^2^* = chi-squared statistic. | | | | | | | |

| Table 2: *Sample Characteristics by Condition (Study 2).* | | | | | | |
| --- | --- | --- | --- | --- | --- | --- |
|  |  | Discrete Condition (*n* = 60) | Mixed Condition (*n* = 59) | *t* | *χ^2^* | p-value |
| Age |  |  |  | 1.50 |  | .135 |
|  | *M* | 20.30 | 19.68 |  |  |  |
|  | *SD* | 4.31 | 2.54 |  |  |  |
| Core Disgust Propensity | |  |  | -0.88 |  | .381 |
|  | *M* | 33.21 | 33.99 |  |  |  |
|  | *SD* | 7.75 | 7.47 |  |  |  |
| Contamination-Based OCD | |  |  | -0.80 |  | .427 |
|  | *M* | 9.36 | 6.87 |  |  |  |
|  | *SD* | 10.01 | 7.18 |  |  |  |
| Sex Assigned at Birth | |  |  |  | 1.98 | .576 |
|  | Female | 108 (75.0%) | 119 (79.3%) |  |  |  |
|  | Male | 30 (20.8%) | 28 (18.7%) |  |  |  |
|  | Non-binary | 5 (3.5%) | 3 (2.0%) |  |  |  |
|  | Prefer Not to Say | 1 (0.7%) | 0 (0%) |  |  |  |
| Race |  |  |  |  | 4.19 | .651 |
|  | White/Caucasian | 123 (85.4%) | 120 (80.0%) |  |  |  |
|  | Black/African American | 9 (6.3%) | 17 (11.3%) |  |  |  |
|  | Asian | 5 (3.5%) | 7 (4.7%) |  |  |  |
|  | American Indian/Alaska Native | 1 (0.7%) | 1 (0.7%) |  |  |  |
|  | Other | 3 (2.1%) | 1 (0.7%) |  |  |  |
| Ethnicity |  |  |  |  | 0.11 | .743 |
|  | Hispanic/Latino | 3 (2.1%) | 4 (2.7%) |  |  |  |


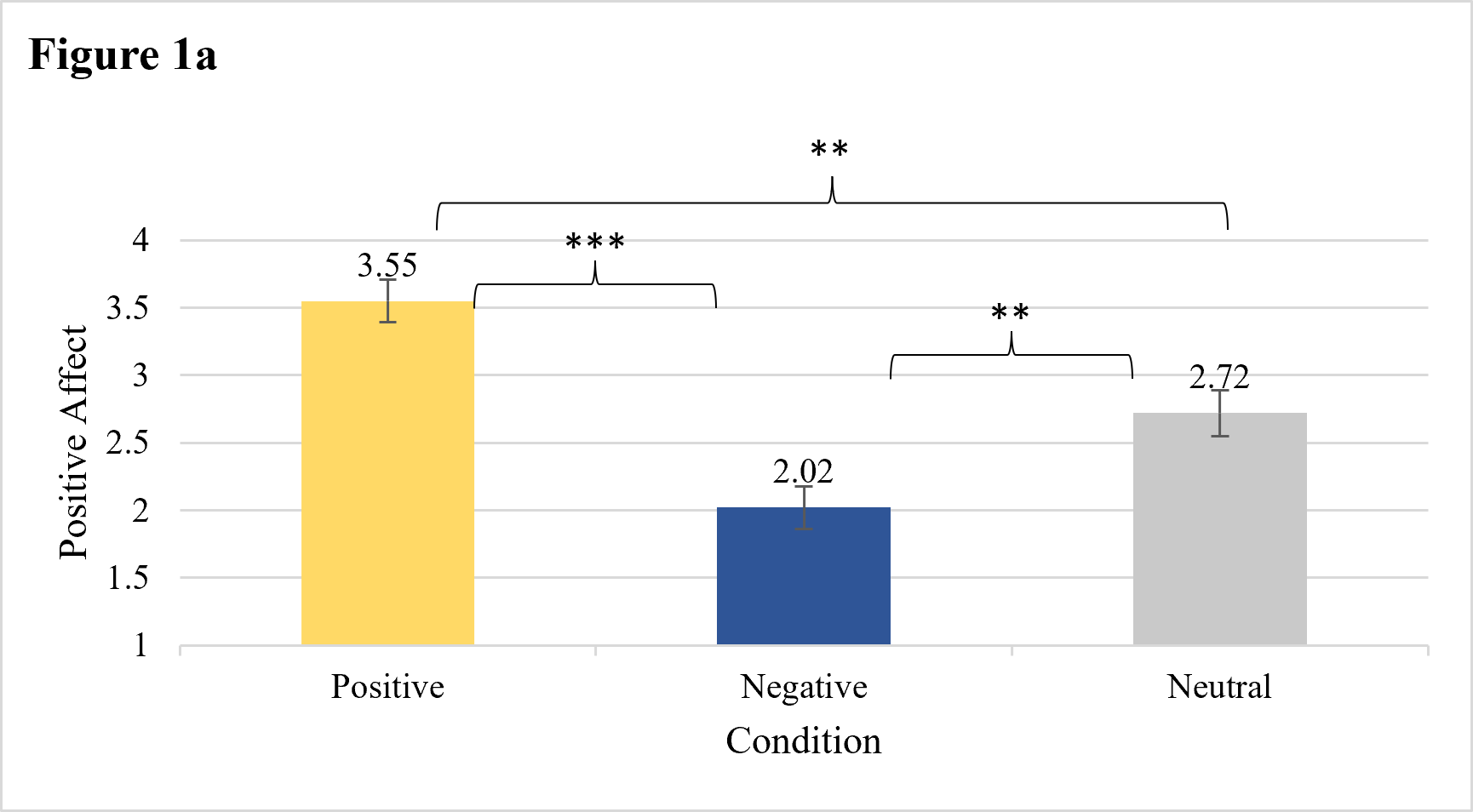

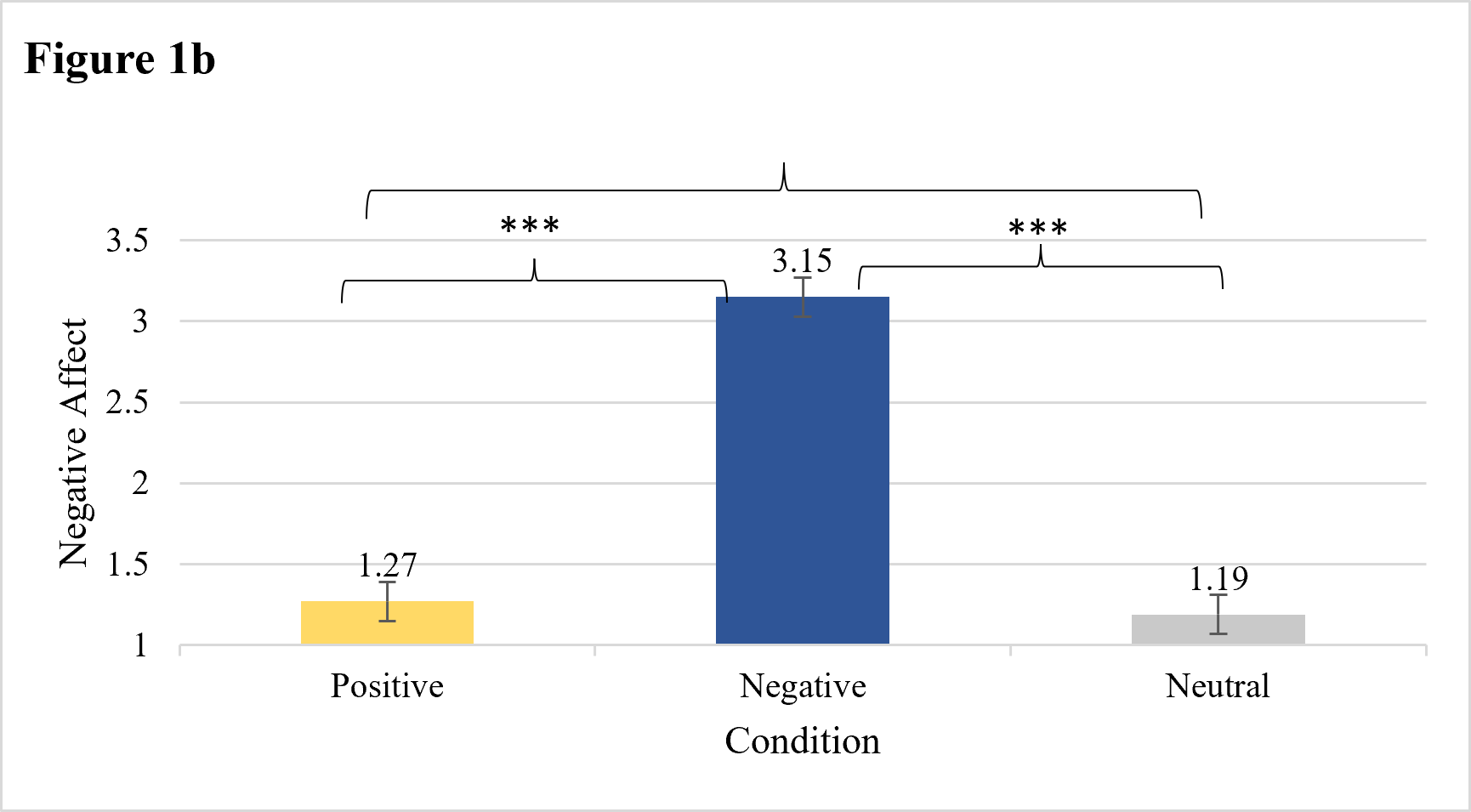


**Figure S1a: Positive Affect Manipulation Check (Study 1)**

**Figure S1b: Negative Affect Manipulation Check (Study 1)**
